# Electroacupuncture alleviates damage to myopic RGCs probably through lncRNA-XR_002789763.1-mediated mitophagy

**DOI:** 10.1186/s13020-025-01058-5

**Published:** 2025-02-02

**Authors:** Xuejun Wang, Qinghong Lin, Li Tian, Xiaoying Li, Teruko Fukuyama, Weijung Ten, Xiehe Kong, Yanting Yang, Xiaopeng Ma, Xingtao Zhou

**Affiliations:** 1 Eye Institute and Department of Ophthalmology, Eye & ENT Hospital, Fudan University, Shanghai, 200031 China; 2 NHC Key Laboratory of Myopia and Related Eye Diseases; Key Laboratory of Myopia and Related Eye Diseases, Chinese Academy of Medical Sciences, Shanghai, 200031 China; 3 Shanghai Research Center of Ophthalmology and Optometry, Shanghai, 200031 China; 4 Refractive Surgery Department, Bright Eye Hospital, Fuzhou, 350001 China; 5 Longhua Hospital, Shanghai University of Traditional Chinese Medicine, Shanghai, 200032 China; 6 Yueyang Hospital of Integrated Traditional Chinese and Western Medicine, Shanghai University of Traditional Chinese Medicine, Shanghai, 200083 China; 7 Shanghai Research Institute of Acupuncture and Meridian, Shanghai University of Traditional Chinese Medicine, Shanghai, 200032 China

**Keywords:** Myopia, Mitophagy, LncRNA, Retinal ganglion cell, LncRNA-XR_002789763.1/miR-342-5p/PINK1 axis

## Abstract

**Background:**

Mitophagy is closely related to the regulation of retinal ganglion cell (RGC) structure and function. Our previous study suggested that long noncoding RNAs (lncRNAs) can cause damage to myopic RGCs. However, whether electroacupuncture (EA) treatment can delay myopia progression through lncRNA-mediated mitophagy in RGCs is currently unknown. This study aimed to investigate the effect of EA on lncRNA-mediated mitophagy in myopic RGCs.

**Methods:**

Our study investigated the modulatory effect of EA on mitophagy in RGCs of guinea pigs with form-deprived myopia (FDM). RNA sequencing was performed to further analyze the expression profiles of lncRNAs and mRNAs in RGCs of guinea pigs with FDM after EA treatment, and the related competing endogenous RNA (ceRNA) network was constructed. Importantly, PINK1, a mitophagy-related gene, was included in the core ceRNA network to explore the relationship between lncRNAs and mitophagy in myopic RGCs regulated by EA. We also collected eyeballs from myopic and highly myopic adults to further verify the mechanistic results.

**Results:**

This study demonstrated that EA treatment delayed the reduction in refraction and increase in axial length and alleviated RGC damage in guinea pigs with FDM. We further found that EA could induce mitophagy in guinea pig RGCs with FDM by promoting the mitophagy-related PINK1/Parkin signaling pathway. Moreover, mitophagy is inhibited in the retina of highly myopic adults. RNA sequencing revealed that 599 lncRNAs and 455 mRNAs were differentially expressed in guinea pig RGCs with FDM after EA treatment. A core ceRNA network was constructed by incorporating PINK1 and verified by related molecular experiments, and we found that EA treatment may induce mitophagy and attenuated RGC injury in guinea pigs with FDM by sponging miR-342-5p through lncRNA-XR_002789763.1 to activate the PINK1/Parkin signaling pathway and promote Mfn2 ubiquitination.

**Conclusion:**

EA treatment might regulate lncRNA-XR_002789763.1/miR-342-5p axis and activate the mitophagy-related PINK1/Parkin signaling pathway, and promote Mfn2 ubiquitination, thereby alleviating RGC damage and delaying myopia progression.

**Supplementary Information:**

The online version contains supplementary material available at 10.1186/s13020-025-01058-5.

## Introduction

In the past few decades, the prevalence of myopia has been on the rise globally, and this condition has become a global public health problem [1, 2]. There were 262 million cases of myopia globally in 2020, and this number is projected to reach 4758 million by 2050 [3]. In recent years, the prevalence of myopia among children and adolescents in China has gradually increased with increasing age and education level [4, 5]. Studies have shown that genetic and environmental factors are major contributors to the development of myopia [6–8].

The retina is composed of a variety of cells, including neurons, glial cells, and retinal ganglion cells (RGCs). Melanopsin- and rod/cone-driven signals in RGCs affect refraction by affecting the corneal radius of curvature and axial length (AL) [9]. Recent studies have shown that abnormal function and a decreased number of RGCs may be closely related to the occurrence and development of myopia [10–12]. Our previous study revealed that myopia can reduce the number and increase apoptosis of RGCs [13]. Mitochondrial autophagy-dependent reprogramming of glycolytic metabolism benefits RGC development [14]. When mitochondrial autophagy is disrupted, the thickness of the RGC layer is significantly reduced, and RGC apoptosis is increased [15]. Promoting mitochondrial autophagy in RGCs can reduce apoptosis and decrease mitochondrial fragmentation, thus increasing the survival of RGCs [16]. However, it is still unknown whether mitophagic dysfunction occurs in myopic RGCs.

**Fig. 1:**
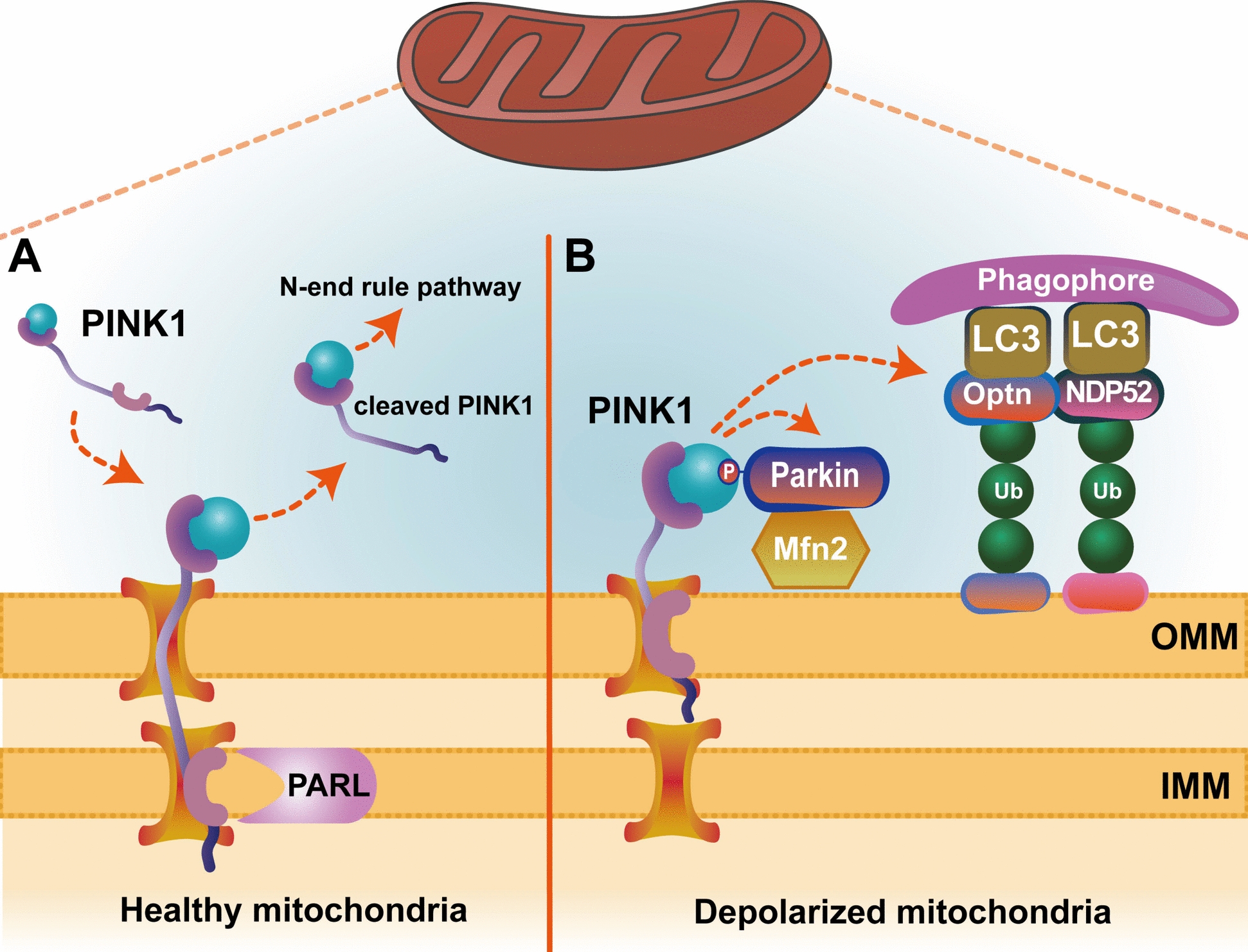
PINK1-dependent mitophagy. **A** Under healthy conditions, PINK1 is inactivated by PARL cleavage at the IMM, followed by degradation via the N-end rule pathway. **B** Mitochondrial depolarization leads to the accumulation of PINK1 in the OMM, which subsequently recruits Parkin, ubiquitinates Mfn2, and recruits the autophagy receptors Optn and NDP52 to mitochondria. Finally, LC3-positive phagocytes undergo mitophagy, which degrades mitochondria through lysosomes. OMM: outer mitochondrial membrane; IMM: inner mitochondrial membrane

The PTEN-induced putative kinase protein 1 (PINK1)/Parkin pathway is essential for promoting cellular mitochondrial autophagy [17]. Under healthy conditions, PINK1 localizes to the mitochondria and translocates to the inner mitochondrial membrane (IMM), where it is inactivated by cleavage by the IMM protease presenilin-associated rhomboid-like protein (PARL) (Fig. 1A) [18]. However, when mitochondria depolarize, PINK1 aggregates in the outer mitochondrial membrane (OMM) and subsequently recruits and phosphorylates Parkin [19]; then, leaded to the ubiquitination of the substrate protein mitofusin 2 (Mfn2) on the mitochondrial membrane (Fig. 1B) [16]. Moreover, the autophagy receptor optineurin (Optn) and nuclear dot protein 52 kDa (NDP52) are recruited to mitochondria, and subsequently, microtubule-associated protein 1 light chain 3 (LC3)-positive phagocytes are formed, which undergo mitophagy (Fig. 1B). Abnormal mitophagy prevents the elimination of damaged mitochondria, which can lead to cell death [20]. However, whether the damage to myopic RGCs is caused by dysfunctional mitochondrial autophagy is still unknown.

Long noncoding RNAs (lncRNAs), through special molecular allosteric effects, can act as a skeleton to recruit transcription factor (TF)-targeted combinations and participate in complex intracellular regulatory mechanisms to mediate the occurrence and development of myopia [21]. LncRNAs may also contribute to the pathogenesis of myopia through G protein-coupled receptor and retinol metabolism [22]. As a competitive endogenous RNAs (ceRNAs), lncRNAs can compete with microRNAs (miRNAs) through miRNA response elements (MREs) to indirectly regulate the expression of related target genes [23, 24]. Our previous study reported that lncRNA-XR_002792574.1, a ceRNA, targeted the miR-760-3p/Adcy1 pathway to cause RGC damage of myopic guinea pigs [13].

Electroacupuncture (EA) has been proven to be an effective treatment for preventing and controlling myopia [25, 26]. Clinically, our previous research revealed that EA can delay the increase in axial length in myopic children and improve retinal surface blood flow [27]. A mechanical study also showed that EA can control the development of myopia by inhibiting the levels of GABA (a retinal inhibitory neurotransmitter) and its receptors of myopic guinea pigs [28]. In addition, it has been shown that EA can control excessive glucocorticoid-enhanced myopia by increasing the resting-state functional connectivity between the left and right visual cortices [26]. Recent studies have also found that EA can reduce apoptosis of ciliary muscle cells in myopic guinea pigs by inhibiting mitochondrial signaling pathway [29]. For the retina, EA can improve retinal function in myopic guinea pigs by inhibiting the RhoA/ROCK2 signaling pathway [30]. However, it remains unclear whether EA can control the development of myopia through lncRNAs in RGCs and whether EA regulates mitophagy in myopic RGCs through lncRNAs.

In this study, we investigated whether the function of mitophagy is impaired in the RGCs of guinea pigs with form deprivation myopia (FDM) and the effect of EA on mitophagy in the RGCs of guinea pigs with FDM. Next, we determined the expressions of lncRNAs and mRNAs in the RGCs of FDM guinea pigs after EA treatment by RNA sequencing, and PINK1 was included for further ceRNA network analysis to explore the relationship between EA-regulated lncRNAs and mitochondrial autophagy. We further collected eyeball samples from patients with myopia or high myopia to verify the underlying mechanism involved. Our study aimed to provide new insights into the pathogenesis of myopia and the molecular mechanism by which EA delays myopia progression by analyzing lncRNA-mediated mitochondrial autophagy in myopic RGCs.

## Materials and methods

### Animals

The 2-week-old male colored guinea pigs used in this study were obtained from Danyang Changyi Experimental Animal Breeding Co., Ltd. (Danyang, China; license no: SYXK (Shanghai) 2018-0040). The animals were kept in the Animal Experiment Center of Yueyang Hospital of Integrated Traditional Chinese and Western Medicine, Shanghai University of Traditional Chinese Medicine. This study was approved by the Laboratory Animal Ethics Committee of Yueyang Hospital of Integrated Traditional Chinese and Western Medicine, Shanghai University of Traditional Chinese Medicine (No. YYLAC-2022-158-1). The experiment followed the statement of the Association for Research in Vision and Ophthalmology (ARVO) regarding the use of animals in research. The experimental animals were housed with a diurnal cycle of 12 h, temperature of 22–24 °C, relative humidity of 40–60%, and brightness of approximately 200 lx. A free diet was provided.

### Induction of the FDM guinea pig model

The guinea pigs were divided into normal control group (NCG), FDM group (FDMG) and electroacupuncture group (EAG), with 21 guinea pigs in each group. All eyes in the NCG were untreated. According to previous studies, the right eyes of both the FDMG and the EAG were covered with a latex balloon as a translucent blindfold for 8 weeks to induce FDM guinea pig model, and the opposite eyes were not covered as a control [31]. Throughout the experiment, we ensured that the right eye was completely covered but could blink freely. After 8 weeks of form deprivation, the refraction and axial Length (AL) of guinea pigs were measured to determine whether the model of FDM was successfully established.

### EA treatment

The guinea pigs of EAG were treated by EA stimulation at bilateral acupoints Fengchi (GB20) and Taiyang (EX-HN5) for 30 min once daily for 4 consecutive weeks in the fifth week of modeling. The movement of the guinea pigs was restricted with a guinea pig immobilization device and their head and eyes were fully exposed for EA intervention based on previous experimental studies in animals [28]. Acupuncture needles and acupoints were wiped with 75% alcohol before EA. Stainless steel acupuncture needles (13 mm × 0.25 mm; Suzhou Medical Appliance Factory, Suzhou, Jiangsu Province, China) were inserted at Fengchi (GB20) and Taiyang (EX-HN5) bilaterally to a depth of 2–3 mm. The handle of the needle was further connected to an electrical stimulator, whose parameters were set as 30 min of EA treatment, continuous electrical pulses, 2 Hz in frequency and 2 mA in intensity. All guinea pigs were awake during the EA treatment. Acupoint localization was referenced to previous studies and published ‘‘Experimental Acupuncturology’’ [28, 32].

### Assessment of refraction and axial length (AL)

Guinea pigs were treated with 0.5% compound tropicamide solution (Santen Pharmaceutical Co., Ltd., Shiga Plant., China) for mydriasis, and refraction was detected in the dark with a streak retinoscope (YZ24; 66 Vision Tech Co., Ltd., China) [33]. We take the average refractive index of the horizontal and vertical meridiens as the final spherical equivalent (SE) refractive index. Measurements were made three times for each eye and averaged. We used A-scan ultrasonography (KN-1800; Kangning, Jiangsu Province, China) to measure the axial length (AL) of guinea pigs [34]. One drop of 0.4% oxytetracaine hydrochloride (Benoxil; Santen, Osaka, Japan) was used to each guinea pig eye before testing, after which ultrasound measurements were performed. The AL of each guinea pig eye was measured ten times in duplicate to calculate the mean value.

### Hematoxylin and eosin (H&E) staining

The pathological morphology of the eyeballs of guinea pigs and humans was observed via H&E staining. The eye tissue was fixed in 4% paraformaldehyde, subjected to gradient dehydration, paraffin embedding, slicing, and drying at 60 °C on a slide, after which HE staining was performed. Finally, the eye sections were sealed and then viewed with an optical microscope (Olympus Corporation, Japan).

### Isolation and culture of RGCs and fluorescence identification

RGCs were extracted from guinea pig eyeballs as described previously [35, 36]. The retina was isolated from guinea pig eyeballs and crushed with ophthalmic scissors in 4 °C ice-cold Hank’s balanced salt solution (HBSS, Invitrogen). The isolated retinal fragments were digested in a solution containing crude collagenase (Sigma), papain (Sigma), 0.02% bovine serum albumin (BSA) (Sigma), and l-cysteine for 30 min at 37 °C. The digested retinal fragments were transferred to OV-1 solution and ovomucoid (Sigma) and ground, and a single-cell suspension was obtained through a 20 µm aperture screen. Culture dishes were pretreated with goat anti-rabbit IgG (HL) antibodies, and the obtained single-cell suspensions were transferred to pretreated culture dishes overnight. The cells were again transferred to culture dishes containing goat anti-mouse IgG + IgM (HL) antibodies. The liquid in the culture dish was then discarded, and the iCell primary neuronal cell culture system (iCell Bioscience, Inc.) was used. Finally, RGCs were seeded on cell culture dishes coated with poly-L-lysine and mouse laminin. According to our previous study [13], the TUJ1 antibody (Beyotime, Shanghai, China) was used for RGC immunofluorescence identification (*Supplementary Fig. 1A*).

### RGC damage assessment

The level of RGC damage was assessed by lactate dehydrogenase (LDH) assay kit (Beyotime, Shanghai, China) and TUNEL staining. For LDH analysis, the primary cells extracted from each group were seeded into 96-well cell culture plates. After 36 h of culture, the cell supernatant was aspirated, and the samples were determined immediately. The absorbance values at 490 nm were measured with a microplate reader (BioTek, USA).

For TUNEL analysis, the retinal sections from guinea pigs and humans were dewaxed and hydrated, the tissue slices were washed with PBS 3 times, and then, the membrane was disrupted by incubation with 0.1% Triton X-100 solution for 15 min at room temperature. Then, the TUNEL reaction solution was prepared, in which the TDT enzyme, dUTP, and buffer were mixed with a ratio of 1:5:50. The sections were incubated with TUNEL reaction solution for 2 h at 37 °C in the dark. The cell nuclei were stained with DAPI and finally sealed with an antifluorescence quenching sealer. Images were acquired using a fluorescence microscope (Olympus Corporation, Japan).

### Donor ocular tissues

The eyeballs of healthy, myopic and highly myopic adults aged 34–65 years were obtained from Bright Eye Hospital (Shanghai, China). The donor eye tissues were excluded from syndromic connective tissue diseases (including congenital cataract, corneal opacity, metabolic cataract, etc.), advanced eye diseases (including age-related macular degeneration and glaucoma, etc.), or severe systemic diseases (such as cancer and diabetes). The eye cups were removed within 5 h after the death of the donor, and the separated retina, choroid and sclera were placed in a storage tube and stored at − 80 °C. All ocular tissues were obtained from Asian populations with balanced sex distributions. This study was approved by the Ethics Committee of Eye & ENT Hospital, Fudan University (No. 2023114) and followed the guidelines of the Declaration of Helsinki.

### RNA isolation and construction of the RNA library

High-throughput sequencing of guinea pig RGCs (Oebiotech, Shanghai, China) was performed to determine lncRNA and mRNA expressions [37]. Three samples from the EAG were tested. Total RNA was extracted by an isolation kit (mirVana™ miRNA Isolation Kit (Thermo)). Next, we measured RNA concentration by NanoDrop-2000 (Thermo), and RNA integrity was assessed using a gel imaging system (Tanon 2500, Biotanon Co., Ltd.). After that, mRNA and lncRNA libraries were constructed from TruSeq-stranded total RNA with a Ribo-Zero Gold Prep Kit (Illumina). After rRNA removal, RNA was purified using magnetic beads. First- and second-strand cDNAs were synthesized, and the library fragments were purified using AMPure XP beads for cDNA screening.

### Bioinformatic analysis of mRNAs and lncRNAs

We compared the RNA sequencing data of RGCs from guinea pigs with FDM after EA treatment with the RNA sequencing data of RGCs from our previous guinea pigs with FDM [13]. The reference genomes of guinea pigs were aligned using HISAT2 [38]. StringTie software was used to determine the fragments for each gene. The transcripts with coding potential were selected by Pfam, CPC, CNCI and PLEK to obtain predicted lncRNA sequences.

The sequencing reads of each sample were aligned with lncRNAs, mRNA transcripts, and predicted lncRNA sequences via Bowtie2. Gene quantitative analysis was conducted by eXpress, and the fragments per kilobase of exon model per million reads mapped (FPKM) values and counts were obtained. DESeq (2012) R package was used to analyze the differentially expressed (DE) lncRNAs and DE mRNAs. Statistical significance was defined as *P* values < 0.05 and |log2(FC)| values > 1. Gene Ontology (GO) and Kyoto Encyclopedia of Genes and Genomes (KEGG) enrichment analyses were performed by hypergeometric distribution tests.

### LncRNA-miRNA-mRNA network construction

By combining miRNAs from the miRanda database, we predicted potential regulation relationships between lncRNAs and miRNAs and between miRNAs and mRNAs.

The score between ceRNA relationship pairs was calculated using the ceRNA MuTATE method, and the probability of miRNAs sharing between ceRNA relationship pairs was calculated using the hypergeometric distribution algorithm. Finally, ceRNA relationship pairs with high reliability were obtained. Cytoscape software was used for ceRNA network construction.

### Quantitative real-time PCR (qRT‒PCR)

Total RNA was extracted from guinea pig RGCs and human retinas by TRIzol reagent (Invitrogen, Carlsbad, CA, USA). The cDNA synthesis of lncRNAs and mRNAs was performed using the PrimeScript RT Reagent Kit with gDNA Eraser (TaKaRa, Japan), and the cDNA synthesis of miRNA was performed using an EZ-press microRNA Reverse Transcription Kit (EZBioscience, USA). The cDNA was amplified on a Roche Light Cycler 480 II using TB Green™ Premix Ex Taq™ (Tli RNaseH Plus) (TaKaRa, Japan) for lncRNAs and mRNAs and using an EZ-press microRNA qPCR Kit (EZBioscience, USA) for miRNAs. The relative amount of RNA expression was analyzed by the 2^−ΔΔCt^ method. GAPDH and U6 were used as controls. The guinea pig and human sequences of primers used are listed in *Supplementary Tables 1–2*. The primers were obtained from the Shanghai Generay Biotech Co., Ltd.

### Western blot (WB)

Total protein was extracted from guinea pig RGCs and the human retina. The same amount of protein was separated by SDS‒PAGE and subsequently transferred to a PVDF membrane (Millipore, Boston, MA, USA). The PVDF membrane was blocked with 5% BSA. Subsequently, primary antibodies against Mfn2 (sc-100560, Santa Cruz, MA, USA), Optn (sc-166576, Santa Cruz, MA, USA), LC3B (ab192890, Abcam, Cambridge, UK), p62 (ab109012, Abcam, Cambridge, UK), PINK1 (ab186303, Abcam, Cambridge, UK), Parkin (ab77924, Abcam, Cambridge, UK), PARL (sc-514836, Santa Cruz, MA, USA) and β-Actin (AF2811, Beyotime, Shanghai, China) were incubated with the PVDF membranes overnight. On the second day, the membranes were incubated with secondary antibody (A0216, A0208, 1:1000; Beyotime, Shanghai, China). Protein was detected by an enhanced chemiluminescence (ECL) kit (Beyotime, Shanghai, China). Protein gray values were examined using ImageJ software.

### Immunofluorescence (IF) and MitoTracker staining

Sections of the eyeball were deparaffinized, and antigen retrieval was performed. The slices were blocked with 5% BSA for 30 min. Primary antibodies against PINK1 (GB114934, Servicebio, Wuhan, China) and LC3A/LC3B (sc-398822, Santa Cruz, MA, USA) were added to the sections, and the sections were incubated overnight at 4 °C in a black wet box. After washing several times in PBS, the sections were incubated with secondary antibodies in a black wet box for 60 min at room temperature. Mitochondria were stained with 200 nM MitoTracker^®^ Red CMXRos (9082S, CST, Danvers, MA, USA) at 37 °C for 30 min. Then, the slices were incubated with DAPI staining solution for 10 min at room temperature. Finally, photos were taken under a fluorescence microscope (Olympus Corporation, Japan). The IOD intensity was analyzed using ImageJ software.

### Coimmunoprecipitation (Co-IP)

Coimmunoprecipitation analysis was performed using the IP/Co-IP kit (Thermo Fisher, Waltham, USA). Primary guinea pig RGCs were lysed with IP lysate containing protease inhibitors for 30 min on ice. The protein concentration was determined by the BCA assay. Then, the antibodies were added, and the samples were incubated for 2 h at 24 °C. The immunoprecipitates were incubated with Protein A/G Plus Agarose at 4 °C. Then, the protein complexes were washed and eluted with Lane Marker Sample Buffer containing 10 mM DTT at 100 °C for 10 min. The eluate was collected after centrifugation. The samples were subjected to SDS‒PAGE and transferred to PVDF membranes for subsequent western blot analysis.

### Immunohistochemistry

For immunohistochemical staining of human samples, eye sections were incubated with an anti-LC3A/LC3B antibody (sc-398822, Santa Cruz, MA, USA) overnight at 4 °C and then sections were incubated with a secondary antibody for 50 min. Subsequently, human eye sections were visualized using a DAB kit for color development. Finally, the human eye sections were stained with hematoxylin for 3 min, and images were acquired using a light microscope.

### Statistical analysis

Statistical analysis was performed with SPSS 25.0 statistical software (IBM, Armonk, NY, USA) and GraphPad Prism 10 (GraphPad Software, San Diego, CA, USA). All the data are expressed as the mean ± standard error of the mean (SEM). Student’s t test was performed for differences between two groups, and one-way ANOVA was performed for differences among three or more groups. *P* < 0.05 was considered to indicate statistical significance.

## Results

### Changes in refraction and axial length in guinea pigs with FDM after EA treatment

**Fig. 2:**
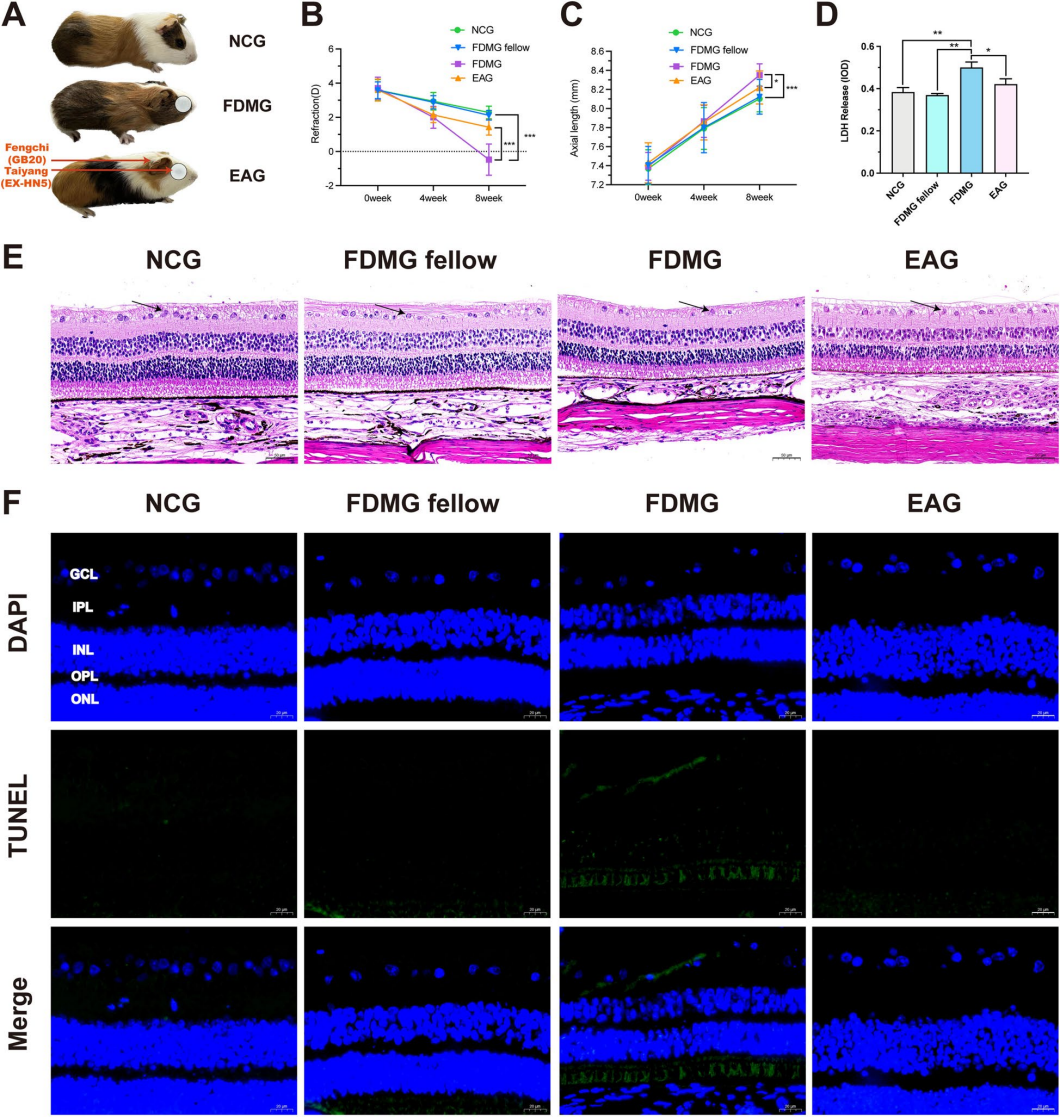
EA treatment alleviated myopia progression and RGC damage in guinea pigs. **A** Photographs of guinea pigs in each group. **B** Refractions of guinea pigs in each group (n = 21). **C** Axial length of guinea pigs in each group (n = 21). **D** cytotoxic LDH release in RGCs from guinea pigs. **E** Histology of the retina stained with H&E in guinea pigs; arrows indicate ganglion cell layer sites (scale bar: 50 µm) (n = 5). **F** TUNEL staining of retinas from guinea pigs. DAPI (blue); TUNEL (green) (scale bar: 20 µm) (n = 3). GCL: ganglion cell layer; IPL: inner plexiform layer; INL: inner nuclear layer; OPL: outer plexiform layer; ONL: outer nuclear layer. *P < 0.05; ***P < 0.001. NCG: normal control group; FDMG fellow: form-deprived myopia fellow control group; FDMG: form-deprived myopia group; EAG: electroacupuncture group

For investigation of the effect of electroacupuncture (EA) on myopic guinea pigs, a form-deprived myopic (FDM) guinea pig model was established and treated with EA (Fig. 2A). The acupoints are shown in Fig. 2A. After 8 weeks of induction, form deprivation induced significant myopia in the FDMG (− 0.47 ± 0.91 D) compared with the NCG (+ 2.28 ± 0.36 D) and FDMG fellow (left eyes) (+ 2.11 ± 0.28 D) (*P* < 0.001) (Fig. 2B). However, compared with the FDMG, the EA treatment significantly delayed the degree reduction (1.41 ± 0.45 D vs. − 0.47 ± 0.91 D, *P* < 0.001) (Fig. 2B). Moreover, compared with those in the NCG (8.10 ± 0.13 mm) and FDMG fellow (8.12 ± 0.18 mm), the axial lengths of the eyes in the FDMG (8.35 ± 0.11 mm) were greater (*P* < 0.001) (Fig. 2C). However, compared with the FDMG, the EA treatment delayed the increase in axial length (8.22 ± 0.17 mm vs. 8.35 ± 0.11 mm, *P* < 0.01) (Fig. 2C). The changes in refractive and axial lengths showed that EA treatment alleviated the progression of myopic guinea pigs.

### EA treatment relieved RGC damage in guinea pigs with FDM

To determine the effect of EA treatment on RGCs in guinea pigs with FDM, we performed LDH, retinal H&E staining and apoptosis assays (Fig. 2D-F). After 8 weeks of induction, compared with the NCG and the FDMG fellow, the FDMG had thinner retinas, an uneven distribution and disordered arrangement of RGCs, increased LDH release in RGCs and TUNEL-positive signals in the GCL (Fig. 2D–F). After EA treatment, although the retina was still thinner, the number of RGCs was normal, and the RGCs were clearly arranged compared with those in the FDMG (Fig. 2E). At the same time EA treatment decreased the LDH release of RGCs in guinea pigs with FDM (*P* < 0.05) (Fig. 2D), and TUNEL staining revealed that RGC apoptosis was lower in the EA treatment group than in the FDMG (Fig. 2F). These results showed that EA treatment can alleviate RGC damage in guinea pigs with FDM.

### EA treatment improved mitophagy in RGCs from guinea pigs with FDM

**Fig. 3:**
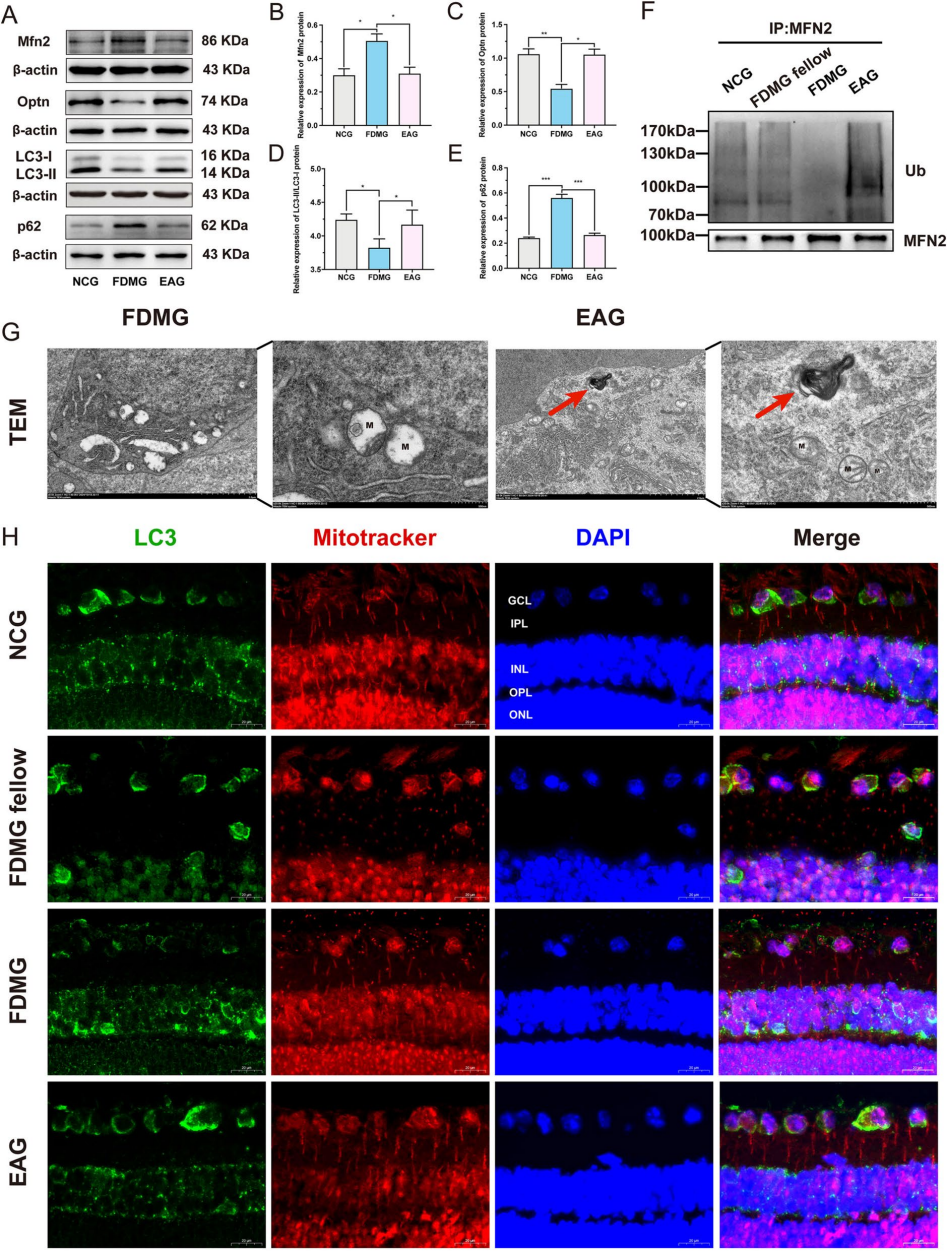
EA treatment induced mitophagy in RGCs from guinea pigs with FDM. **A–E** WB analysis of Mfn2 protein expression (**A, B**), Optn protein expression (**A, C**), LC3-II/I protein expression (**A, D**) and p62 protein expression (**A, E**) in guinea pig RGCs (n = 3). **F** Mfn2 ubiquitination in guinea pig RGCs was detected by coimmunoprecipitation. **G** The ultrastructure of RGCs in FDM guinea pigs was observed by transmission electron microscopy (n = 2), and autophagolysosomes are pointed by red arrows. Magnification: × 8000 and × 20,000; Scale bar: 2 µm and 500 nm; M: mitochondria; TEM: transmission electron microscopy. **H** Guinea pig retinal sections were stained with MitoTracker (red) and immunostained with an LC3 (green) antibody. The colocalization of mitochondria and LC3 was detected by immunofluorescence (scale bar: 20 µm) (n = 5). GCL: ganglion cell layer; IPL: inner plexiform layer; INL: inner nuclear layer; OPL: outer plexiform layer; ONL: outer nuclear layer. *P < 0.05; **P < 0.01; ***P < 0.001. NCG: normal control group; FDMG fellow: form-deprived myopia fellow control group; FDMG: form-deprived myopia group; EAG: electroacupuncture group

To explore whether EA treatment could alleviate RGC damage by promoting mitophagy, we assessed the mitophagy-related proteins Mfn2, Optn, LC3 and p62. First, primary RGCs were isolated from guinea pigs and identified as RGCs by immunofluorescence staining; the RGC-specific marker TUJ1 appeared red (*Supplementary Fig. 1A*). Next, WB analysis revealed that the protein level of Mfn2 was significantly greater in RGCs from the guinea pigs with FDM than in those from the NCG (*P* < 0.05) (Fig. 3A, B). After EA treatment, the expression of the Mfn2 protein in the RGCs of the guinea pigs with FDM was significantly decreased (*P* < 0.05) (Fig. 3A, B). Studies have shown that the ubiquitination of the mitochondrial protein Mfn2 is required for autophagy-related proteins to recognize damaged mitochondria [39]. Therefore, we performed coimmunoprecipitation assays to analyze the level of Mfn2 ubiquitination. In RGCs from the guinea pigs with FDM, we observed a reduced level of Mfn2 protein ubiquitination (Fig. 3F). However, EA promoted Mfn2 protein ubiquitination in the RGCs of the guinea pigs with FDM (Fig. 3F).

Furthermore, WB analysis revealed that compared with those in the NCG, the expression of the mitophagy-related proteins Optn and LC3-II/I in the RGCs from guinea pigs with FDM was decreased (*P* < 0.05) (Fig. 3A, C, D), and p62 expression was significantly increased (*P* < 0.001) (Fig. 3A, E). However, EA treatment significantly increased the protein level of Optn and the ratio of LC3-II to LC3-I (*P* < 0.05) (Fig. 3A, C, D) and decreased the protein level of P62 in the RGCs of the guinea pigs with FDM (*P* < 0.001) (Fig. 3A, E).

In addition, severe mitochondrial (M) damage and broken mitochondrial (M) membrane structure were observed by transmission electron microscopy in the RGCs of FDM guinea pigs, and the typical autophagy structure was not observed (Fig. 3G). However, after EA treatment, the mitochondrial (M) damage in FDM guinea pig RGCs was relatively mild, the mitochondrial (M) membrane structure was relatively intact, and the presence of autophagolysosomes (red arrows) was observed (Fig. 3G). To further analyze mitochondrial autophagy in RGCs, immunofluorescence colocalization of mitochondria (red) and autophagy-related protein LC3 (green) was performed. The results confirmed that impaired mitophagy in the GCL of the guinea pigs with FDM, and EA treatment induced mitophagy in the GCL of the guinea pigs with FDM (Fig. 3H). In summary, these data showed that EA alleviates RGC damage by inducing mitophagy in guinea pigs with FDM.

### EA treatment induced mitophagy in RGCs from guinea pigs with FDM by promoting the PINK1/Parkin signaling pathway

**Fig. 4:**
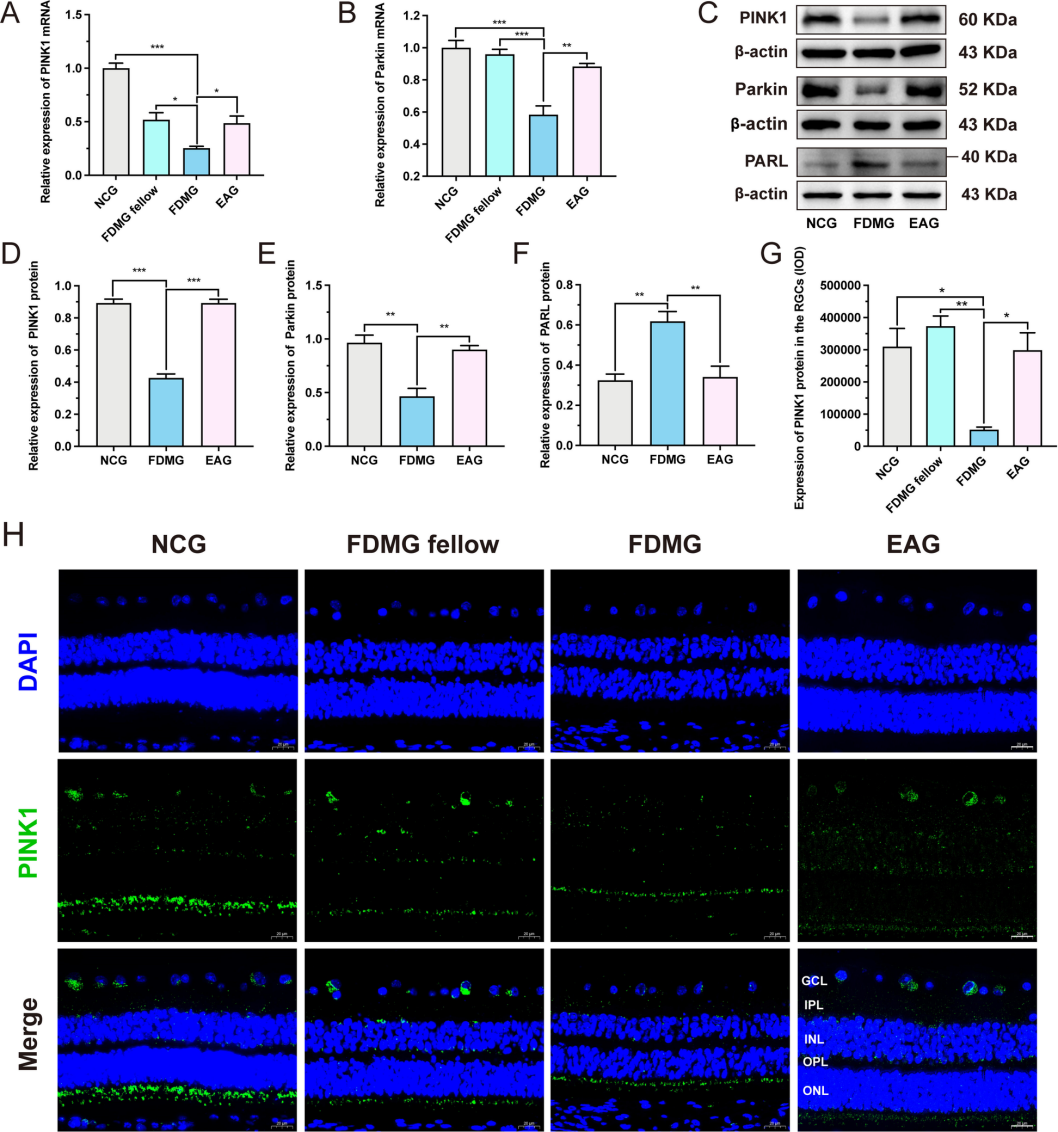
EA treatment induced mitophagy in RGCs from guinea pigs with FDM by promoting the PINK1/Parkin signaling pathway. **A, B** The mRNA expression levels of PINK1 and Parkin in guinea pig RGCs were measured by qRT‒PCR (n = 3‒5). **C–F** WB analysis of PINK1 protein expression (**C, D**), Parkin protein expression (**C, E**) and PARL protein expression (**C, F**) in guinea pig RGCs (n = 3). **G** IOD analysis of PINK1 (green) expression in the GCL of guinea pigs (n = 5). **H** Images of PINK1 (green) immunofluorescence in the retina of guinea pigs with nuclear staining (DAPI: blue) (scale bar: 20 µm) (n = 5). GCL: ganglion cell layer; IPL: inner plexiform layer; INL: inner nuclear layer; OPL: outer plexiform layer; ONL: outer nuclear layer. *P < 0.05; **P < 0.01; ***P < 0.001. NCG: normal control group; FDMG fellow: form-deprived myopia fellow control group; FDMG: form-deprived myopia group; EAG: electroacupuncture group

PINK1/Parkin pathway is currently recognized as the key regulatory mechanism of mitophagy and is essential for the initiation of autophagy [40]. Therefore, we hypothesized that EA-induced mitophagy in RGCs from guinea pigs with FDM may be dependent on the PINK1/Parkin signaling pathway. PCR and WB analysis revealed that the mRNA and protein levels of PINK1 and Parkin were significantly lower in the RGCs from the guinea pigs with FDM than in NCG RGCs (*P* < 0.01) (Fig. 4A-E). However, EA treatment increased the mRNA and protein levels of PINK1 and Parkin in the RGCs of the guinea pigs with FDM (*P* < 0.05) (Fig. 4A–E).

Moreover, the immunofluorescence results further revealed lower PINK1 (green) staining in the GCL of the guinea pigs with FDM than in those of the NCG and FDMG fellow (*P* < 0.05) (Fig. 4G, H), and EA treatment promoted PINK1 (green) staining in the GCL of the guinea pigs with FDM (*P* < 0.05) (Fig. 4G, H). Regarding PARL expression, WB results showed that compared with NCG, the PARL expression was higher in RGCs from the guinea pigs with FDM (*p* < 0.01) (Fig. 4C, F), and EA treatment inhibited PARL expression in the RGCs of the guinea pigs with FDM (*p* < 0.01) (Fig. 4C, F). In summary, these data showed that EA may induce autophagy in RGCs from guinea pigs with FDM by promoting the PINK1/Parkin signaling pathway.

### Screening differentially expressed lncRNAs in RGCs between EA-treated guinea pigs and guinea pigs with FDM

**Fig. 5:**
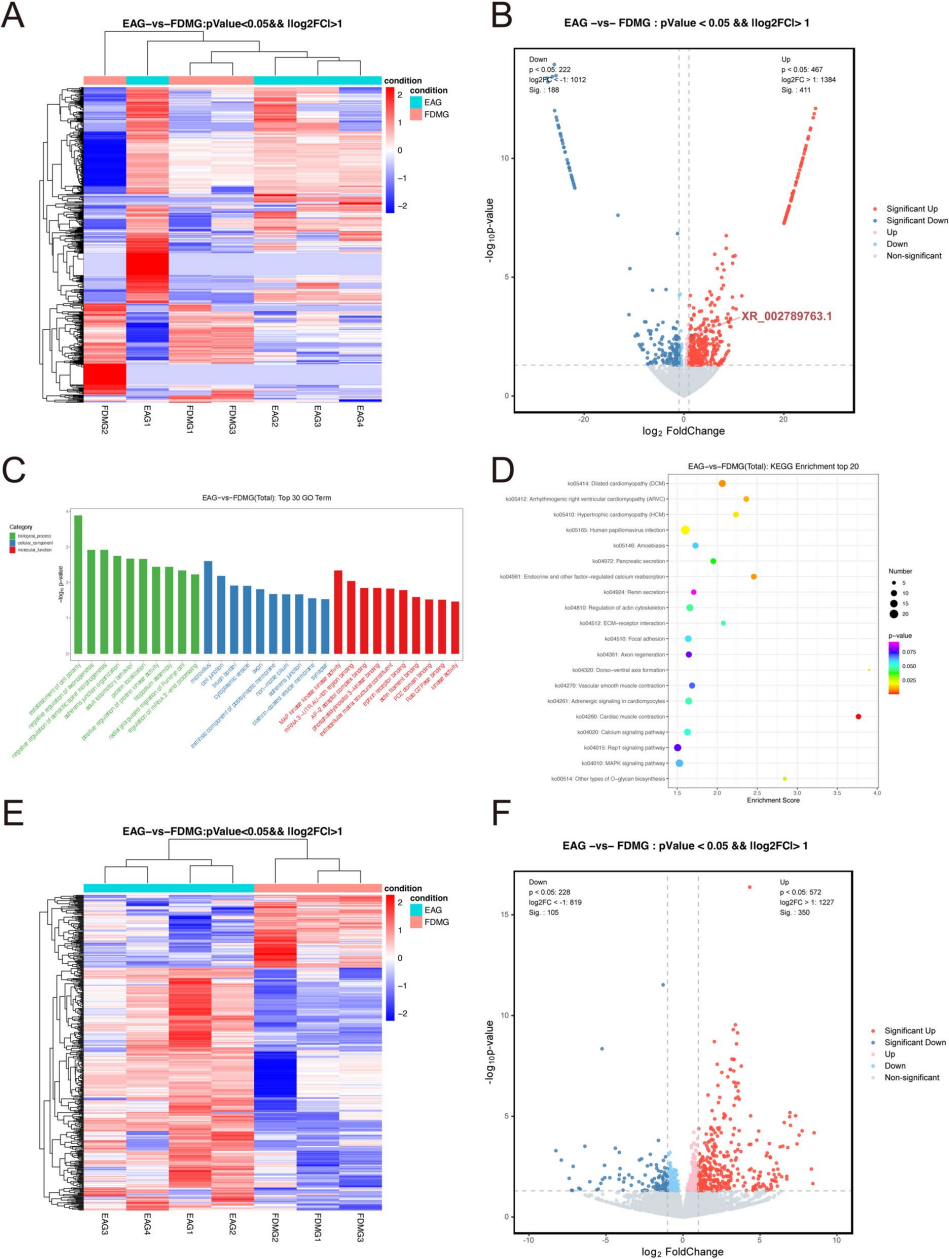
The differentially expressed (DE) lncRNAs and mRNAs were assessed by RNA sequencing in the EAG (n = 4) and FDMG (n = 3). Heatmap (**A**) and volcano plot (**B**) of DE lncRNAs between the EAG and FDMG (log2FC > 1 and *P* < 0.05). **C** Top 30 GO terms for the DE genes between the EAG and FDMG. **D** KEGG enrichment of the top 20 DE genes between the EAG and FDMG. Heatmap (**E**) and volcano plot (**F**) of DE mRNAs between the EAG and FDMG (log2FC > 1 and *P* < 0.05). FDMG: form-deprived myopia group; EAG: electroacupuncture group

To explore the differential changes in the lncRNAs expressions in RGCs of guinea pigs with FDM after EA treatment, we extracted RGCs from guinea pigs with FDM after EA treatment for RNA sequencing and compared them with our previous RNA sequencing data for RGCs of guinea pigs with FDM (Sequence Read Archive, https://www.ncbi.nlm.nih.gov/sra/PRJNA1021213). As shown in Fig. 5A and B, there were 411 upregulated and 188 downregulated differentially expressed (DE) lncRNAs in the EAG compared with the FDMG (log2FC > 1, *P* < 0.05). The top 10 most significantly up-regulated and down-regulated lncRNAs between EAG and FDMG are shown in *Supplementary Table 3*.

To further determine the potential roles of the lncRNAs, we performed GO term and KEGG pathway enrichment analyses using the parental genes of the DE lncRNAs (Fig. 5C, D). The top 30 enriched GO terms of the DE lncRNAs in the EAG are presented and included the biological process (negative regulation of axonogenesis and positive regulation of protein kinase activity), cellular component (cytoplasmic vesicle), and molecular function (RNA 3’ − UTR AU − rich region binding and kinase activity) terms (Fig. 5C). The enriched KEGG pathways were related to the ECM − receptor interaction, calcium signaling pathway, MAPK signaling pathway and other types of O-glycan biosynthesis (Fig. 5D). Taken together, these results indicate that the mechanism by which EA alleviates myopia progression is closely related to the expression of lncRNAs in RGCs.

### Screening differentially expressed mRNAs in RGCs between EA-treated guinea pigs and guinea pigs with FDM

Based on our previous RNA sequencing data of RGCs from guinea pigs with FDM, we further explored the DE mRNAs in these cells after EA treatment. A heatmap and volcano plot were generated for the DE mRNAs in the EAG compared to those in the FDMG (log2FC > 1, *P* < 0.05) (Fig. 5E, F). Among the 455 DE mRNAs, 350 were upregulated and 105 were downregulated in the EAG relative to the FDMG. According to the fold change in the mRNAs between the two groups, the top 10 up-regulated and down-regulated mRNAs were screened, and the results are presented in Supplementary Table 4. In addition, GO term and KEGG pathway enrichment analyses were performed on the mRNAs (*Supplementary Fig. 1B, C*).

### LncRNA–miRNA–mRNA network construction

**Fig. 6:**
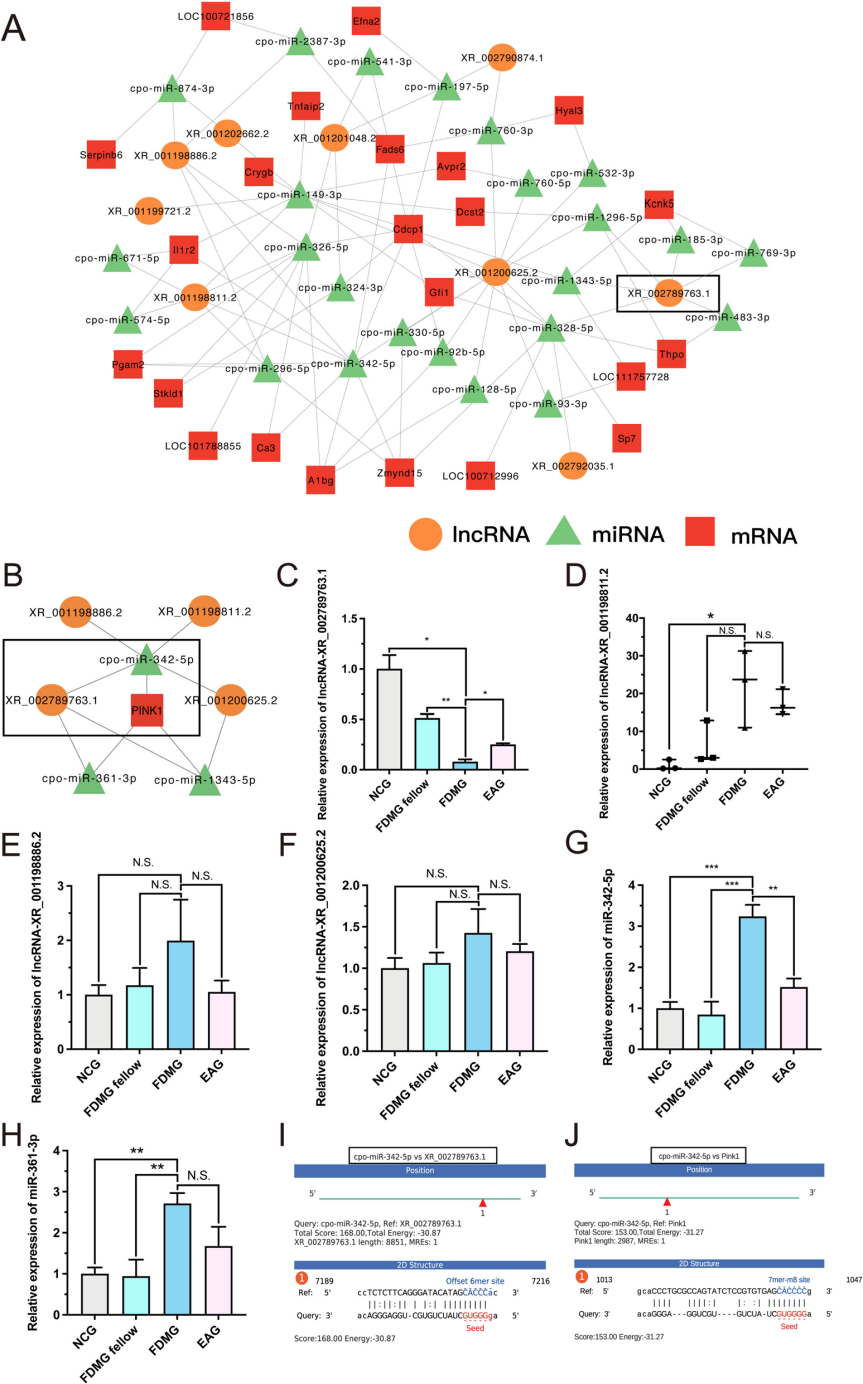
EA treatment activated the PINK1/Parkin signaling pathway by sponging miR-342-5p with lncRNA-XR_002789763.1 in RGCs from guinea pigs with FDM. **A** LncRNA-miRNA-mRNA network analysis between the EAG and FDMG. **B** Analysis of the core lncRNA-miRNA-mRNA network associated with PINK1. Orange, green and red represent lncRNAs, miRNAs and mRNAs, respectively. **C**–**F** The expression levels of lncRNA-XR_002789763.1, lncRNA-XR_001198811.2, lncRNA-XR_001198886.2 and lncRNA-XR_001200625.2 were measured by qRT‒PCR in guinea pig RGCs (n = 3‒6). **G, H** The expression levels of miR-342-5p and miR-361-3p were measured by qRT‒PCR in guinea pig RGCs (n = 3). **I** The targeting relationship between lncRNA-XR_002789763.1 and miR-342-5p was predicted. **J** The targeting relationship between miR-342-5p and PINK1 was predicted. N.S. *P* > 0.05; *P < 0.05; **P < 0.01; ***P < 0.001. NCG: normal control group; FDMG fellow: form-deprived myopia fellow control group; FDMG: form-deprived myopia group; EAG: electroacupuncture group

To further elucidate the potential interactions of DE lncRNAs and mRNAs, we constructed a lncRNA-miRNA-mRNA interaction network based on DE lncRNAs and mRNAs as well as miRNAs base on the miRanda database. A total of 13 coexpressed lncRNAs, 129 coexpressed mRNAs and 656 miRNAs were selected for ceRNA network construction. We analyzed lncRNA-miRNA and miRNA-mRNA regulatory pairs using miRanda v3.3a and identified a total of 94 miRNA‒lncRNA regulatory pairs and 603 miRNA‒mRNA regulatory pairs. Next, according to the results of ceRNA analysis, a ceRNA network of 95 mRNA-miRNA-lncRNA regulatory pairs was constructed out of 30 mRNA-lncRNA pairs (Fig. 6A). Our results showed that EA promoted mitophagy through the PINK1/Parkin signaling pathway in RGCs from guinea pigs with FDM (Figs. 3, 4). To elucidate the important role of the mitophagy-related gene PINK1 in the ceRNA network, we included PINK1 for further ceRNA analysis to construct a core lncRNA‒miRNA-mRNA network (Fig. 6B).

### EA treatment might regulate lncRNA-XR_002789763.1/miR-342-5p/PINK1 axis in RGCs from guinea pigs with FDM

Subsequently, we validated the 4 lncRNAs in the core lncRNA-miRNA-mRNA network by qRT‒PCR. The results showed that lncRNA-XR_002789763.1 expression was significantly lower in RGCs from guinea pigs with FDM compared with NCG and FDMG guinea pig RGCs (*P* < 0.05) (Fig. 6C). However, EA treatment increased the expression of lncRNA-XR_002789763.1 in the RGCs of the guinea pigs with FDM (*P* < 0.05) (Fig. 6C), which was consistent with our RNA sequencing results (Fig. 5B). After EA treatment, the expression of the other 3 lncRNAs (lncRNA-XR_001198811.2, lncRNA-XR_001198886.2 and lncRNA-XR_001200625.2) did not change in RGCs from the guinea pigs with FDM (*P* > 0.05) (Fig. 6D–F). Finally, we further validated the top 2 miRNAs that might interact with lncRNA-XR_002789763.1 and PINK1 mRNA in the ceRNA network (Fig. 6G, H). PCR results showed that both miR-342-5p and miR-361-3p were more highly expressed in RGCs from the guinea pigs with FDM than in NCG and FDMG group guinea pig RGCs (*P* < 0.01) (Fig. 6G, H). However, only miR-342-5p expression was downregulated in the RGCs of the guinea pigs with FDM treated with EA (Fig. 6G). We further used the miRanda v3.3a program to predict the targeting relationships among lncRNA-miRNA-mRNA sequences and found that lncRNA-XR_002789763.1 can bind to miR-342-5p and that miR-342-5p can bind to PINK1 (Fig. 6I, J).

Therefore, based on the above results, we suggest that lncRNA-XR_002789763.1 might be related to PINK1 in RGCs from guinea pigs with FDM. EA treatment can reduce RGC damage and slow myopia progression in guinea pigs with FDM, which may be related to the activation of the mitophagy-associated PINK1/Parkin signaling pathway through lncRNA-XR_002789763.1 sponging of miR-342-5p in these cells.

### Analysis of the regulatory miR-342-5p/PINK1/Parkin axis in adult retinas with high myopia

**Fig. 7:**
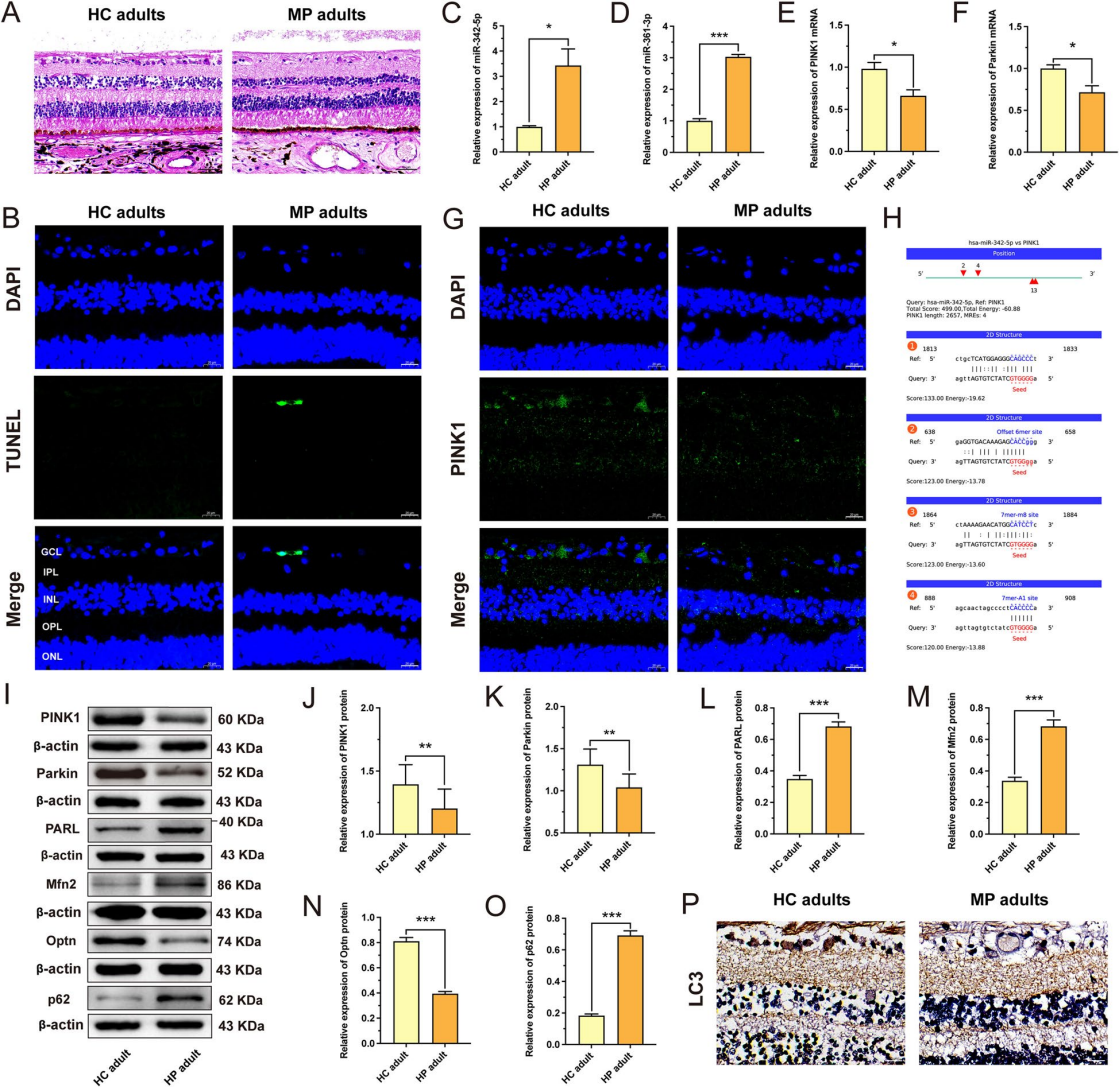
The study of the regulatory miR-342-5p/PINK1/Parkin axis in retinas of adults with high myopia. **A** Histology of the retina stained with H&E in myopic adults (scale bar: 50 µm). **B** TUNEL analysis of retinas from myopic adults. DAPI (blue); TUNEL (green) (scale bar: 20 µm). **C, D** The expression levels of miR-342-5p and miR-361-3p were measured by qRT‒PCR in the retinas of highly myopic adults (n = 3). **E**, **F** The mRNA expression levels of PINK1 and Parkin in the retinas of highly myopic adults were measured by qRT‒PCR (n = 3). **G** Images of PINK1 (green) immunofluorescence in the retinas of myopic adults with nuclei (DAPI: blue) (scale bar: 20 µm). **H** The targeting relationship between miR-342-5p and PINK1 was predicted. **I**–**O** WB analysis of PINK1 protein expression (**I, J**), Parkin protein expression (**I, K**), PARL protein expression (**I, L**), Mfn2 protein expression (**I, M**), Optn protein expression (**I**, **N**), and p62 protein expression (**I, O**) in the retinas of highly myopic adults (n = 3–4). **P** Images of LC3 (brown) immunohistochemistry in the retina of myopic adults (scale bar: 20 µm). GCL: ganglion cell layer; IPL: inner plexiform layer; INL: inner nuclear layer; OPL: outer plexiform layer; ONL: outer nuclear layer. *P < 0.05; **P < 0.01; ***P < 0.001. HC adults: healthy control adults; MP adults: myopic adults; HP adults: highly myopic adults

On this basis, we further collected eyeball samples from myopic and highly myopic adult donors to verify the miR-342-5p/PINK1/Parkin axis. First, H&E and TUNEL staining revealed disarrangement of the retina and RGCs and increased apoptotic signals in the GCL in myopic adults, which was consistent with the results in guinea pigs with FDM (Fig. 7A, B). PCR results also showed that the expression of miR-342-5p and miR-361-3p was significantly increased (*P* < 0.05) (Fig. 7C, D), and the expression of PINK1 and Parkin was significantly decreased (*P* < 0.05) (Fig. 7E, F) in the retinas of highly myopic adults than healthy control adults. Moreover, the immunofluorescence results further demonstrated that the level of PINK1 (green) in the GCL of myopic adults was lesser than that in the GCL of healthy control adults (Fig. 7G). Subsequently, the binding site of human miR-342-5p to PINK1 was predicted using the miRanda v3.3a program. The results showed that miR-342-5p targeted PINK1 (Fig. 7H).

In addition, WB results showed that PINK1/Parkin signaling was inhibited in the highly myopic adult retina (Fig. 7I–K), which was consistent with the mRNA results. Furthermore, the mitophagy-related proteins PARL, Mfn2, Optn and P62 were assessed in the retinas of highly myopic adults by WB (Fig. 7I, L–O), and the findings were consistent with the results obtained for FDM guinea pigs. The immunohistochemical results further revealed that LC3 (brown) staining was lower in the GCLs of myopic adults than in those of healthy control adults (Fig. 7P). In summary, using clinical samples from patients with high myopia, we further revealed that the miR-342-5p/PINK1/Parkin axis may be involved in the development of high myopia and that mitophagy may be inhibited in RGCs in high myopia.

## Discussion

Recent studies have shown that lncRNAs play important roles in the progression of myopia [21, 22]. Our previous study revealed that the lncRNA-XR_002792574.1/miR-760-3p/Adcy1 axis inhibited the cGMP/PKG and apelin signaling pathways in RGCs, resulting in myopia-related RGC damage [13]. The survival of RGCs may be increased by normalizing mitophagy [16, 41]. However, the relationship between lncRNAs and mitophagy in myopic RGCs remains to be studied.

EA has been proven to effectively delay the progression of myopia in guinea pigs [26, 29]. Our previous results further showed that EA could delay the increase in axial length and improve retinal surface blood flow in myopic children [27]. However, whether EA can regulate mitochondrial autophagy through lncRNAs in RGCs and delay the progression of myopia is still unclear. In this study, we examined the regulation of mitophagy by EA in RGCs from guinea pigs with FDM. The DE expression of lncRNAs and mRNAs in RGCs from guinea pigs with FDM after EA treatment were further analyzed, and PINK1 was included in the ceRNA analysis to explore the relationship between lncRNAs and mitophagy. We also collected eyeballs from myopic patients to further verify the above results.

First, we established a guinea pig model of FDM, during which EA was administered (Fig. 2A). Our previous clinical studies have shown that EA at Taiyang, Fengchi and other acupoints can delay the growth of axial length in myopic children, and increase the retinal surface vessel density and retinal surface blood perfusion density [27]. According to the traditional Chinese medicine theory of “selection of acupoints is based on the location of problems” [42]. Taiyang and Fengchi acupoints are located around the eye area, and EA stimulation of these two acupoints can achieve the effect of dredging meridians, promoting blood circulation and brightening the eyes. If blood vessels are blocked, the eye cannot function normally. Therefore, the Taiyang and Fengchi can regulate the local meridian-collaterals, qi and blood of the eye to achieve the purpose of treating myopia. In addition, Fengchi point is the acupoint of foot Shaoyang gallbladder meridian, which has the effect of soothing the liver and brighten the eyes. Animal experiments have also shown that electroacupuncture at Taiyang and other acupoints can reduce the levels of GABA receptors in the retina of myopic guinea pigs, and thus inhibit the axial length growth of myopic guinea pigs [28]. Recent studies have also found that EA at Taiyang and other acupoints can alleviate the progression of myopia in guinea pigs by inhibiting mitochondrial apoptosis signaling pathway [29]. Therefore, based on the above, we investigated the mechanism of EA at Taiyang and Fengchi acupoints in delaying the progression of myopia (Fig. 2A).

Our results point out that the refraction of the guinea pigs with FDM shifted toward myopia, and the axial length of the eyes of the FDM guinea pigs increased (Fig. 2B, C). However, EA treatment significantly delayed the degree reduction (Fig. 2B), the axial length result showed that EA treatment deferred the increase in axial length (Fig. 2C). The above results show that, EA treatment delayed the progression of myopia in myopic guinea pigs (Fig. 2B, C), which is consistent with previous studies [26, 29]. We further determined the effect of EA treatment on RGCs from guinea pigs with FDM. H&E and TUNEL staining revealed that the RGCs were unevenly distributed and disordered in the guinea pigs with FDM, with increased apoptotic signaling in the GCL (Fig. 2E, F), which is consistent with the results in myopic patients (Fig. 7A, B). However, after EA treatment, the number of RGCs in the guinea pigs with FDM was normal, and their arrangement was clear (Fig. 2E). One study reported that EA can alleviate the apoptosis of ciliary muscle cells in guinea pigs with lens-induced myopia [29]. Our TUNEL results further showed decreased apoptosis in the GCL of the guinea pigs with FDM after EA treatment (Fig. 2F). We further shown that EA treatment diminished the LDH release of RGCs in guinea pigs with FDM (Fig. 2D). These results suggest that EA treatment relieves RGC damage in FDM guinea pigs.

Studies have shown that there is mitochondrial DNA damage and dysfunction in the lens epithelium of cataract patients with high myopia [43], and impaired mitophagy is an important feature of the pathology of ophthalmic diseases [44]. Therefore, we hypothesized that mitochondrial dysfunction may be an underlying factor with high myopia. Mitochondria are organelles that regulate cell energy and cell death [45], and the removal of damaged mitochondria by mitophagy is essential for the maintenance of cell viability and homeostasis [46–48]. Therefore, we further investigated whether EA treatment attenuated RGC damage in myopic guinea pigs by inducing mitophagy. We extracted primary RGCs in guinea pigs (Supplementary Fig. 1A), and WB analysis revealed that Mfn2 expression was increased in RGCs from guinea pigs with FDM; this increase was reversed by EA treatment (*P* < 0.05) (Fig. 3A, B). The ubiquitination of mitochondrial proteins such as Mfn2 is essential for the recognition of damaged mitochondria by autophagy-related proteins [39]. Our coimmunoprecipitation results showed that EA treatment significantly increased the level of Mfn2 ubiquitination in the RGCs of the guinea pigs with FDM (Fig. 3F). A previous study showed that induced Mfn2 ubiquitination promotes the clearance of damaged mitochondria [49].

Accumulating evidence suggests that OPTN is an autophagy inducer that initiates the autophagic process [50] and that loss of OPTN impairs mitophagic flux in cells [51]. Our results exposed that EA promoted the expression of the autophagy receptor Optn (*P* < 0.05) (Fig. 3A, C). Another study showed that stimulating LC3 protein expression and reducing p62 levels could alleviate mitochondrial damage by normalizing mitophagy and inhibiting mitochondria-mediated apoptosis [52]. Our results suggested that mitophagy is impaired in RGCs from guinea pigs with FDM and that EA treatment ameliorated mitochondrial autophagy in these cells by regulating the mitophagy-related proteins LC3-II/I and p62 (*P* < 0.05) (Fig. 3A, D, E). The transmission electron microscopy and the colocalization of mitochondria with LC3 also further indicated that EA treatment induced mitophagy in the RGCs of guinea pigs with FDM (Fig. 3G, H). In conclusion, EA treatment attenuates RGC damage by inducing mitophagy in guinea pigs with FDM. Moreover, consistent with the results in FDM guinea pigs, mitophagy was impaired in the retinas of myopic and highly myopic grownups (Fig. 7I, M-P).

To date, the molecular mechanism of mitophagy has been extensively studied, with studies mainly focusing on the PINK1/Parkin signaling pathway in response to mitochondrial stress [53, 54]. After its precursor is imported into mitochondria, PINK1 is cleaved in the transmembrane segment by the intramitochondrial membrane protease PARL and is rapidly degraded [18]. Pharmacological blockade of PARL has been shown to promote PINK1/Parkin-dependent mitophagy [55]. Studies have shown that Parkin is recruited to mitochondria via the upregulation of PINK1 [56], followed by the ubiquitination of Mfn2 [49]. The autophagy receptor Optn is then recruited to ubiquitinated mitochondria to form LC3-positive phagophores, thereby initiating mitophagy [57] (Fig. 1). Therefore, we examined whether the promotion of mitophagy by EA in RGCs from FDM guinea pigs is dependent on the PINK1/Parkin signaling pathway. The results showed that the PINK1/Parkin signaling pathway was inhibited and that PARL expression was increased in the RGCs of guinea pigs with FDM (*P* < 0.05) (Fig. 4). As expected, EA promoted PINK1/Parkin signaling and decreased PARL expression in RGCs from guinea pigs with FDM (*P* < 0.05) (Fig. 4). Consistent with the results in guinea pigs with FDM, PINK1/Parkin signaling was also inhibited, and PARL expression was enlarged in highly myopic adult retinas (Fig. 7E, F, I–L). Moreover, immunofluorescence further demonstrated a decreasing trend in PINK1 expression in the GCLs of myopic grownups (Fig. 7G). In summary, EA induced autophagy in RGCs from guinea pigs with FDM by promoting the PINK1/Parkin signaling pathway.

To explore whether EA treatment could regulate myopia development via lncRNAs in RGCs, we extracted RGCs from guinea pigs with FDM after EA treatment for RNA sequencing and compared the results with our previous RNA sequencing data from guinea pigs with FDM [13]. We found that 411 DE lncRNAs were up-regulated and 188 DE lncRNAs were down-regulated in RGCs from guinea pigs with FDM after EA treatment (Fig. 5A, B). GO analysis revealed that the DE lncRNAs in the guinea pigs with FDM after EA treatment were mainly associated with the positive regulation of protein kinase activity and kinase activity (Fig. 5C). The regulation of mitogen-activated protein kinase phosphorylase-1 activity was shown to attenuate retinal photoreceptor apoptosis induced by blue light [58]. Other studies have shown that the PI3K/Akt pathway is closely related to pathological myopia [59].

The enriched KEGG pathways were mainly linked to ECM–receptor interactions, the calcium and the MAPK signaling pathway and other types of O-glycan biosynthesis (Fig. 5D). Our previous study also identified KEGG-enriched ECM–receptor interactions and mucin type O–glycan biosynthesis for DE lncRNAs in RGCs from guinea pigs with FDM [13]. Another study also indicated that imbalances in the calcium and MAPK signaling pathway are key pathways involved in lens changes in high myopia [60]. Therefore, the above results suggest that the mechanism by which EA treatment delays myopia progression is closely related to lncRNAs in RGCs.

Based on the RNA sequencing data, we further identified 455 DE mRNAs in the EAG relative to those in the FDMG: 350 up-regulated and 105 down-regulated mRNAs (Fig. 5E, F). In addition, GO term and KEGG pathway enrichment analyses were performed on the mRNAs (*Supplementary Fig. 1B, C*). Studies have shown that lncRNAs involved in gene regulation are called ceRNAs, and lncRNAs affect mRNA expression through competitive binding with miRNAs [61–63]. Therefore, to further determine the potential function of the lncRNAs regulated by EA in guinea pigs with FDM, we constructed a ceRNA network (Fig. 6A).

Our studies have shown that EA promotes mitochondrial autophagy in RGCs from FDM guinea pigs via the PINK1/Parkin signaling pathway (Figs. 3, 4). To elucidate whether EA treatment can regulate mitophagy through lncRNAs in RGCs from guinea pigs with FDM, we included PINK1 as a ceRNA for further analysis and constructed a core lncRNA-miRNA-mRNA network (Fig. 6B). Subsequently, we validated 4 lncRNAs in the core lncRNA-miRNA-mRNA network (Fig. 6B). The results reported that lncRNA-XR_002789763.1 was significantly decreased in RGCs from FDM guinea pigs, and this effect was reversed by EA treatment (*P* < 0.05) (Fig. 6C). Finally, we verified the top 2 miRNAs in the ceRNA network that might interact with lncRNA-XR_002789763.1 and PINK1 mRNA (Fig. 6G, H). The results showed that both miR-342-5p and miR-361-3p were highly expressed in RGCs from guinea pigs with FDM (*P* < 0.01) (Fig. 6G, H). As expected, the changes in the expression of miR-342-5p and miR-361-3p in the retinas of highly myopic grownups were consistent with those in the retinas of guinea pigs with FDM (Fig. 7C, D). However, only miR-342-5p expression was downregulated in RGCs from EA-treated guinea pigs with FDM (*P* < 0.01) (Fig. 6G, H). Our previous study also demonstrated that miR-342-5p was significantly increased in RGCs of guinea pigs with FDM and that miR-342-5p was closely related to myopia development [13]. Using the miRanda v3.3a program, we further found that lncRNA-XR_002789763.1 could target miR-342-5p and that miR-342-5p could target PINK1 (Figs. 6I, J, 7H).

**Fig. 8:**
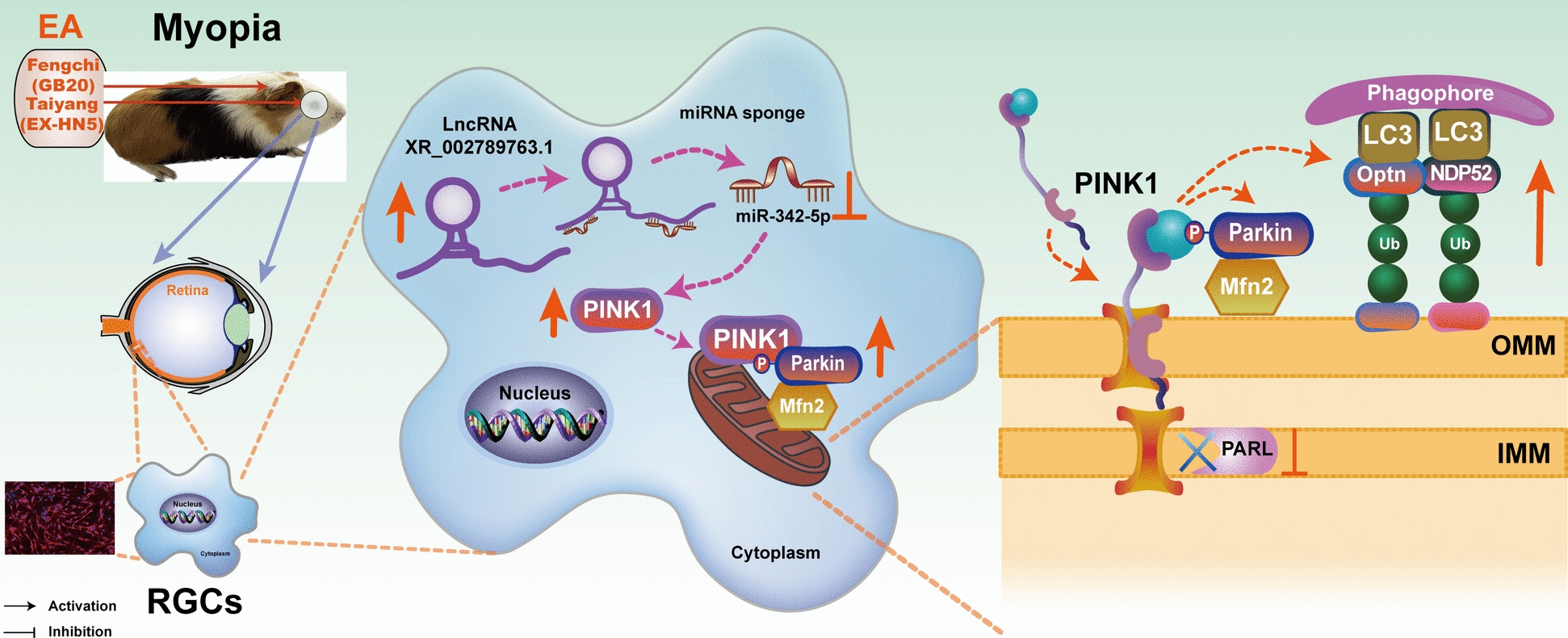
EA treatment might activate the mitophagy-related PINK1/Parkin signaling pathway through lncRNA-XR_002789763.1 sponging of miR-342-5p to promote Mfn2 ubiquitination, thereby inducing mitophagy and reducing RGC damage in myopic guinea pigs. EA: electroacupuncture; RGCs: retinal ganglion cells; OMM: outer mitochondrial membrane; IMM: inner mitochondrial membrane

Therefore, the above results suggest that EA treatment might regulate lncRNA-XR_002789763.1/miR-342-5p axis and activate the mitophagy-related PINK1/Parkin signaling pathway, and promote Mfn2 ubiquitination, thereby reducing RGC damage and delaying myopia progression in guinea pigs (Fig. 8). However, this study has several potential limitations. First, we found that the expression of lncRNA-XR_002789763.1 is reduced in RGCs from FDM guinea pigs. However, whether gene knockout or knockdown of lncRNA-XR_002789763.1 can cause spontaneous myopia is still unknown. In future studies, we will further clarify whether gene knockout or knockdown of lncRNA-XR_002789763.1 can induce spontaneous myopia in guinea pigs or mice, and we will investigate whether EA can also activate the mitophagy-related PINK1/Parkin signaling pathway after inhibiting lncRNA-XR_002789763.1. Second, cell transfection experiments should be performed in future studies to further verify the relationships between lncRNA-XR_002789763.1 and miR-342-5p and between miR-342-5p and PINK1. Finally, we will further examine other relevant experiments on the effects of EA on visual function and retinal function in future studies.

## Conclusion

In summary, we determined the expression profiles of lncRNAs and mRNAs regulated by EA in RGCs from guinea pigs with FDM and included the mitophagy-related PINK1 gene in a ceRNA network analysis. Eyeballs from patients with myopia or high myopia were also collected to further verify the above results. We suggest that lncRNA-XR_002789763.1 may be involved in the occurrence and development of myopia by regulating RGC damage through the miR-342-5p/PINK1 pathway. EA treatment might regulate lncRNA-XR_002789763.1/miR-342-5p axis and activate the mitophagy-related PINK1/Parkin signaling pathway, and promote Mfn2 ubiquitination, thereby alleviating RGC damage and delaying myopia progression in guinea pigs (Fig. 8). Overall, our findings provide new insights into the molecular mechanisms by which EA delays the progression of myopia.

## Supplementary Information

Supplementary material 1.

## Data Availability

All data generated or analysed during this study are included in this published article.

## Abbreviations

lncRNA: Long non-coding RNA
miRNA/miR: MicroRNA
RGCs: Retinal ganglion cells
ceRNA: Competing endogenous RNA
FDM: Form-deprived myopia
GO: Gene ontology
KEGG: Kyoto Encyclopedia of Genes and Genomes
EA: Electroacupuncture
AL: Axial length
PINK1: PTEN-induced putative kinase protein 1
IMM: Inner mitochondrial membrane
PARL: Presenilin-associated rhomboid-like protein
OMM: Outer mitochondrial membrane
Mfn2: Mitofusin 2
Optn: Optineurin
LC3: Microtubule-associated protein 1 light chain 3
IF: Immunofluorescence
Co-IP: Coimmunoprecipitation
PBS: Hosphate‐buffered saline
qRT-PCR: Quantitative real time polymerase chain reaction
H&E: Hematoxylin and Eosin
WB: Western blot
DE: Differentially expressed
