# Supplementary material for: Electroacupuncture alleviates damage to myopic RGCs probably through lncRNA-XR_002789763.1-mediated mitophagy
Source: Chin Med. 2025 Feb 2;20:16. doi: 10.1186/s13020-025-01058-5 (PMC11787754; doi:10.1186/s13020-025-01058-5)
Supplement: Supplementary file 1 — Supplementary material 1. [file 13020_2025_1058_MOESM1_ESM.docx]

**Supplementary materials**

*Supplementary Table 1 primer sequences of guinea pig.*

| Gene | Primer F | Primer R |
| --- | --- | --- |
| Cpo-lncRNA-XR_002789763.1 | 5'CGACTTGGCGACTTGCTCTT 3' | 5'TTTCACAAACCAGCAGCGAC3' |
| Cpo- lncRNA-XR_001198811.2 | 5'TCTCCCTCGTCTGGCTACTG3' | 5'ATGTGCATAGGGCTGGTGTG3' |
| Cpo-lncRNA-XR_001198886.2 | 5'TTTTGCCGCTAGAGATGCTG 3' | 5'CCAGGTCCACGTCAGAACATT3' |
| Cpo- lncRNA-XR_001200625.2 | 5'GCTTTCCCAAAATGGGCCTG3' | 5'AGGTGGGGCAAAGCAAAAAC 3' |
| Cpo-miR-342-5p | 5'AGGGGTGCTATCTGTGCTGG3' | - |
| Cpo-miR-361-3p | 5'GGTCCCCCAGGTGTGATTCT3' | - |
| Cpo-PINK1 | 5'GCAGTTGCGTACAGAAAATCCA3' | 5'CTCACACGGAGATACTGGCG3' |
| Cpo-Parkin | 5'GATTTAACCCGCGACAACCG  3' | 5 GCTCCTTCCCCGCAAAAATC  '3' |
| Cpo-Gapdh | 5'GCTGATGCCCCTATGTTCGT 3' | 5'GGATGCGGGGATGATGTTCT3' |
| U6 | 5' CCTGCTTCGGCAGCACA3 3' | - |

*Supplementary Table 2 primer sequences of human.*

| Gene | Primer F | Primer R |
| --- | --- | --- |
| hsa-miR-342-5p | 5'AACAAGAGGGGTGCTATCTGTGATT 3' | - |
| hsa-miR-361-3p | 5'AACAAGTCCCCCAGGTGTGATT 3' | - |
| hsa-PINK1 | 5' CCCAAGCAACTAGCCCCTC 3' | 5'GGCAGCACATCAGGGTAGTC 3' |
| hsa-Parkin | 5'GTGTTTGTCAGGTTCAACTCCA 3' | 5'GAAAATCACACGCAACTGGTC 3' |
| hsa-Gapdh | 5'AAAATCAAGTGGGGCGATGC 3' | 5'TGGTTCACACCCATGACGAA 3' |
| U6 | 5' CCTGCTTCGGCAGCACA 3' | - |

*Supplementary Table 3 top 10 most significantly upregulated and downregulated DE lncRNAs between the EAG and FDMG.*

| Gene ID | Gene symbol | Fold Change | Log2 FC | *P* | | Trend |
| --- | --- | --- | --- | --- | --- | --- |
| TCONS_00032405 | - | 85397122.5 | 26.34768412 | | 7.88E-13 | UP |
| TCONS_00102681 | - | 71256347.09 | 26.08651519 | | 1.32E-12 | UP |
| TCONS_00032555 | XLOC_022630 | 62079278.43 | 25.88760845 | | 1.95E-12 | UP |
| TCONS_00015137 | XLOC_010699 | 43353631.65 | 25.36964951 | | 5.30E-12 | UP |
| TCONS_00034162 | - | 40770853.66 | 25.28103483 | | 6.28E-12 | UP |
| TCONS_00048455 | - | 32067751.64 | 24.93461987 | | 1.21E-11 | UP |
| TCONS_00046605 | XLOC_032628 | 29814904.52 | 24.82953038 | | 1.47E-11 | UP |
| TCONS_00015830 | XLOC_011166 | 29751194.4 | 24.82644425 | | 1.48E-11 | UP |
| TCONS_00034632 | XLOC_024169 | 29688739.66 | 24.82341251 | | 1.49E-11 | UP |
| TCONS_00085566 | XLOC_060277 | 23203424.91 | 24.46783443 | | 2.89E-11 | UP |
| TCONS_00090889 | XLOC_064071 | 5.53E-09 | -27.43089339 | | 4.18E-14 | DOWN |
| TCONS_00077351 | XLOC_054518 | 6.24E-09 | -27.25590439 | | 6.05E-14 | DOWN |
| TCONS_00098026 | XLOC_069267 | 1.15E-08 | -26.37785292 | | 3.62E-14 | DOWN |
| TCONS_00106931 | XLOC_075913 | 1.51E-08 | -25.97753512 | | 1.11E-14 | DOWN |
| TCONS_00091536 |  | 1.60E-08 | -25.90116265 | | 9.77E-13 | DOWN |
| TCONS_00043614 | XLOC_030379 | 1.91E-08 | -25.64162686 | | 3.29E-14 | DOWN DOWN |
| TCONS_00031185 |  | 1.99E-08 | -25.58003502 | | 1.85E-12 | DOWN |
| TCONS_00024827 | XLOC_017337 | 2.21E-08 | -25.43131351 | | 2.48E-12 | DOWN |
| TCONS_00066853 | XLOC_047122 | 2.61E-08 | -25.19248656 | | 3.96E-12 | DOWN |
| TCONS_00052585 | XLOC_037115 | 2.75E-08 | -25.11516746 | | 4.61E-12 | DOWN |
|  |  |  |  |  | |  |

DE: differentially expressed; FC: fold change; FDMG: form-deprived myopia group; EAG: electroacupuncture group.

*Supplementary Table 4 top 10 most significantly upregulated and downregulated DE mRNAs between the EAG and FDMG.*

| Gene ID | Fold Change | Log2 FC | *P Value* | Trend |
| --- | --- | --- | --- | --- |
| Sprr1b | 365.9069504 | 8.51533301 | 6.59E-05 | UP |
| Agbl4 | 346.0888827 | 8.434998789 | 0.021852995 | UP |
| Il1a | 323.2782794 | 8.336632768 | 0.004145218 | UP |
| Pvrig | 213.0368921 | 7.734959477 | 5.26E-05 | UP |
| Pkd1l1 | 186.2830913 | 7.541352918 | 8.60E-05 | UP |
| Mmrn1 | 169.5367389 | 7.405454132 | 0.000232916 | UP |
| LOC100715777 | 158.952617 | 7.312452959 | 9.37E-06 | UP |
| Tespa1 | 126.2243998 | 6.979847007 | 2.81E-05 | UP |
| Kcnk5 | 123.2458981 | 6.945395821 | 6.56E-06 | UP |
| Pou4f2 | 121.3480918 | 6.923007612 | 1.05E-05 | UP |
| LOC100716044 | 0.003276822 | -8.253486802 | 0.000509786 | DOWN |
| LOC106029085 | 0.004185269 | -7.900463818 | 0.001495028 | DOWN |
| Hao1 | 0.00595582 | -7.391484225 | 0.011957977 | DOWN |
| Dnmt3l | 0.006623295 | -7.238235223 | 0.046433143 | DOWN |
| Speradb | 0.006911873 | -7.176707563 | 0.048335032 | DOWN |
| LOC111755242 | 0.00713414 | -7.131044686 | 0.00304705 | DOWN DOWN |
| LOC111755931 | 0.009213692 | -6.762004988 | 0.037085832 | DOWN |
| Erich6b | 0.009806258 | -6.672081516 | 0.041404119 | DOWN |
| LOC100735529 | 0.012087243 | -6.370371024 | 0.000307302 | DOWN |
| LOC100736121 | 0.0150959 | -6.049699386 | 0.01767372 | DOWN |
|  |  |  |  |  |

DE: differentially expressed; FC: fold change; FDMG: form-deprived myopia group; EAG: electroacupuncture group.

*Supplementary Figure 1* *Identification of primary RGCs and* GO and KEGG enrichment analysis of the DE mRNAs between EAG and FDMG.


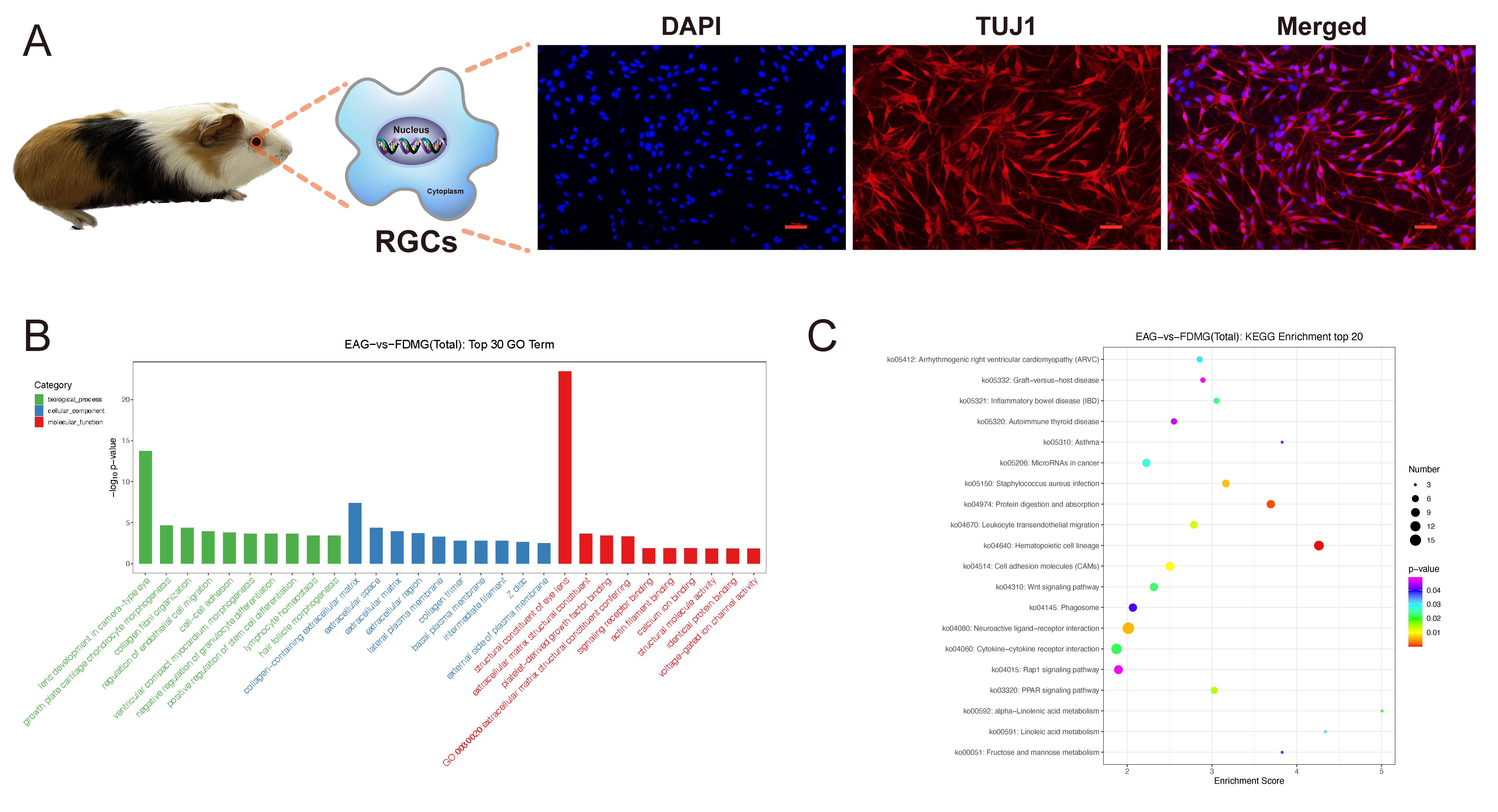


Supplementary Figure 1 (A) Identification of primary RGCs in guinea pigs. DAPI (blue); TUJ1 (red) (scale bar: 50 μm). (B) top 30 GO terms from the mRNA enrichment analysis and (C) top 20 significant KEGG pathways between EAG and FDMG. DE: differentially expressed; FDMG: form-deprived myopia group; EAG: electroacupuncture group.
